# The inhibitory effect of 6-gingerol and cisplatin on ovarian cancer and antitumor activity: *In silico*, *in vitro*, and *in vivo*

**DOI:** 10.3389/fonc.2023.1098429

**Published:** 2023-03-03

**Authors:** Zohreh Salari, Ahmad Khosravi, Elham Pourkhandani, Elaheh Molaakbari, Ehsan Salarkia, Alireza Keyhani, Iraj Sharifi, Hadi Tavakkoli, Samira Sohbati, Shahriar Dabiri, Guogang Ren, Mohammad Shafie’ei

**Affiliations:** ^1^ Obstetrics and Gynecology Center, Afzalipour School of Medicine, Kerman University of Medical Sciences, Kerman, Iran; ^2^ Leishmaniasis Research Center, Kerman University of Medical Sciences, Kerman, Iran; ^3^ Department of Chemistry, Shahid Bahonar University of Kerman, Kerman, Iran; ^4^ Department of Clinical Science, School of Veterinary Medicine, Shahid Bahonar University of Kerman, Kerman, Iran; ^5^ Afzalipour School of Medicine and Pathology and Stem Cells Research Center, Kerman University of Medical Sciences, Kerman, Iran; ^6^ School of Engineering and Computer Science, University of Hertfordshire, Hatfield, United Kingdom; ^7^ Faculty of Medicine, Kerman University of Medical Sciences, Kerman, Iran

**Keywords:** ovarian neoplasm, gingerol, cisplatin, apoptosis, angiogenesis, molecular dynamics simulation, chick embryo

## Abstract

**Background:**

Epithelial ovarian cancer is very common in women and causes hundreds of deaths per year worldwide. Chemotherapy drugs including cisplatin have adverse effects on patients’ health. Complementary treatments and the use of herbal medicines can help improve the performance of medicine. 6-Gingerol is the major pharmacologically active component of ginger. In this study, we compared the effects of 6-gingerol, cisplatin, and their combination in apoptotic and angiogenetic activities *in silico*, in test tubes, and in *in vivo* assays against two ovarian cancer cell lines: OVCAR-3 and human umbilical vein endothelial cells (HUVECs).

**Methods:**

The drug-treated cell lines were evaluated for their cytotoxicity, cell cycle, and apoptotic and angiogenetic gene expression changes.

**Results:**

The proportion of apoptosis treated by 6-gingerol coupled with cisplatin was significantly high. In the evaluation of the cell cycle, the combination therapy also showed a significant promotion of a higher extent of the S sequence. The expression of p53 level, Caspase-8, Bax, and Apaf1 genes was amplified again with combination therapy. Conversely, in both cell lines, the cumulative drug concentrations reduced the expression of VEGF, FLT1, KDR, and Bcl-2 genes. Similarly, in the control group, combination treatment significantly decreased the expression of VEGF, FLT1, KDR, and Bcl-2 genes in comparison to cisplatin alone.

**Conclusions:**

The findings of the present study demonstrated that the cisplatin and 6-gingerol combination is more effective in inducing apoptosis and suppressing the angiogenesis of ovarian cancer cells than using each drug alone.

## 1 Introduction

Cancer is the most prevalent cause of death worldwide. Ovarian malignancy is the third most common gynecological cancer in women after cervical cancer (1). This cancer has the worst prognosis and the highest mortality rate (2, 3). Although it is less common than breast cancer, it is three times more deadly and is projected to increase dramatically by 2040 (1). The high mortality of this cancer is because its growth is secretive and asymptomatic until the end stages of the disease (4). That is why it is called the silent killer (2).

More than half of ovarian cancers have local recurrences and eventually extrapulmonary metastases that are non-surgical and require systemic and palliative care at this stage. Chemotherapy is a systemic treatment for advanced cancer that lowers the size of the tumor and minimizes the symptoms of the disease. Numerous studies have shown that the use of chemoradiotherapy before surgery increases the complete pathological response of the tumor, which is one of the critical factors in the prognosis of the disease (5–7).

Cisplatin is among the most widely applied drugs that can be used alone or combined with other chemotherapy drugs such as paclitaxel, carboplatin, topotecan, etoposide, and doxorubicin in the treatment of most solid tumors, including ovarian cancer. It reacts with the nitrogen atoms of adenine and guanine in the DNA molecule of the cancer cells, causing DNA damage and blocking cell division, and eventually apoptosis or cell death. However, despite the initial efficacy of this drug, its long-term administration not only causes drug resistance (8) but also results in side effects such as neutropenia, anemia, and thrombocytopenia (5, 9).

Angiogenesis, which involves the production of new blood vessels in the growth areas of new tissues, is a normal physiological phenomenon that occurs in conditions such as wound healing or fetal growth. This phenomenon also occurs in cases of mass tumor expansion. There are many genes involved in angiogenesis, of which one of the most important is VEGF-A. Increased mRNA of this factor has been observed in malignant ovarian cancer cells (10, 11).

In recent decades, researchers have sought to find herbal compounds that, in addition to having no side effects, can be used as adjunctive drugs in addition to chemotherapy to treat cancer. Studies show that vegetable oils have an anticancer role and can effectively treat cancer with an anti-inflammatory effect (12).

Ginger is one of the plants containing phenolic compounds that have been used in various cultures, especially in Iran, for various uses, including cooking as a spice and in traditional medicine. This plant has different components, including 3-gingerol, 6-gingerol, 3-shogaols, 6-shogaols, and paradole, among which 6-gingerol is the most active metabolite of ginger, which has a broad range of pharmacological properties such as anti-nausea, anticoagulant, antimicrobial, antioxidant, anti-inflammatory, and anticancer properties. Among 6-gingerols, 6-gingerol-6 is the most pharmacologically active metabolite (13, 14).

Studies showed that 6-gingerol had a greater anti-angiogenic and apoptotic effect than those of other components of ginger. It has an inhibitory effect on the growth of cancerous breast, ovary, pancreas, prostate, and intestine tumors (15, 16) while 6-gingerol slowed the progression of skin cancer cells in mice by preventing the induction of p53 proto-oncogene (17). 6-Gingerol can also prevent the proliferation of different types of cancers including HPV-infected cells in the cervix, reactivates the apoptotic factor p53, and accelerates DNA destruction by cancer cells. Interestingly, 6-gingerol also induces the expression of apoptotic-associated genes including Caspase-3 and PARP, and reduces tumor volume (18–21)

The foundation for docking conformations was the binding affinity of 6-gingerol and cisplatin with apoptotic (Bax, Bcl-2, and Caspase-8) and angiogenetic (VEGF-A and KDR1) mediator genes (22). Considering the anti-inflammatory effect of 6-gingerol and its positive effects on cancer cell lines, we decided to investigate a wide range of experimentation to assess the effect of the substances on their apoptosis and angiogenesis against ovarian cancer *in silico*, *in vitro*, and *in vivo* models using a chick model in compositions of cisplatin alone and their combination.

## 2 Materials and methods

### 2.1 *In silico* modeling

#### 2.1.1 Ligand and target/receptor preparation

**Figure 1:**
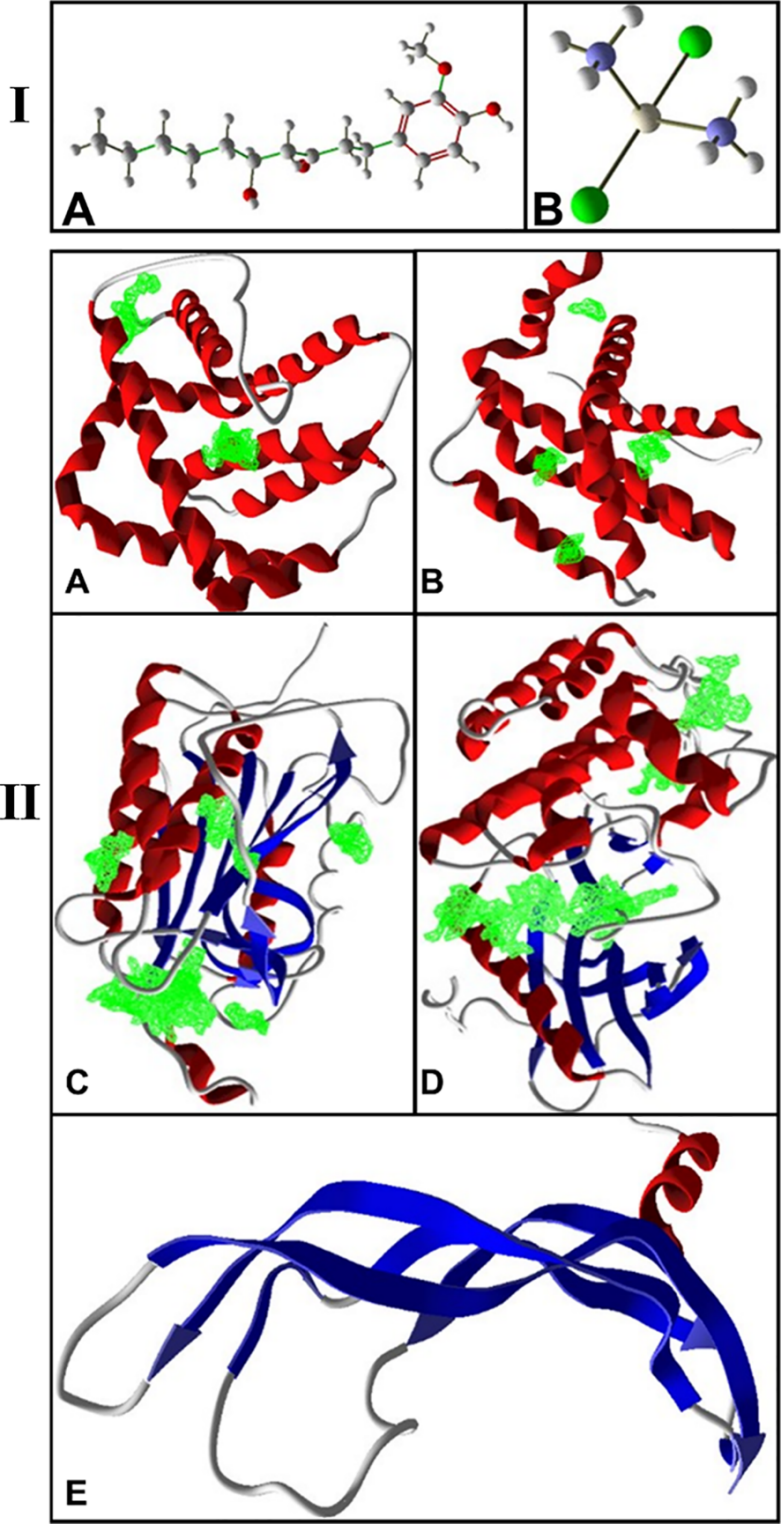
**(I)** 3D structure of **(A)** 6-gingerol and **(B)** cisplatin as stick and bond types in a position using MVD studies. **(II)** Docking configuration and dynamic sites of **(A)** Bax, **(B)** Bcl-2, **(C)** Caspase-8, **(D)** KDR1, and **(E)** VEGF-A target proteins (PDB ID: 5W5X, 5JSN, 1I4E, 2QU5, and 5T89, respectively) using MVD studies.

The conformers of 6-gingerol and cisplatin in three dimensions and sdf format (**Figures 1 IA, B**) were retrieved from the PubChem compounds database at the National Center for Biotechnology Information (NCBI) website (www.pubchem.ncbi.nlm.nih.gov). KDR 1 and VEGF-A as the consistent angiogenic-regulating genes and Bax and Caspase-8 as apoptotic-regulator proteins were chosen as our receptors in this investigation. At first, the target protein structures of Bax, Caspase-8, KDR1, and VEGF-A targets (PDB ID: 5W5X, 5JSN, 1I4E, 2QU5, and 5T89, respectively) were obtained from the RCSB Protein Data Bank (https://www.rcsb.org/) (**Figures 1 IIA-E**). Subsequently, to prepare a structure for molecular docking, supplementary factors in the PDB file were removed using MVD software.

#### 2.1.2 Molecular docking (MVD) process

The precision of the AutoDock Vina package is approximately 80%, more advanced than that of AutoDock 4.2 (23), while this is close to 87% for the MVD software (24). The structure of protein and compounds was organized, employing the “preparation molecule for docking” unit of MVD; then, cavities of protein were detected as appropriate poses on the receptor for ligand binding. A maximum iteration of 1,500, a grid resolution of 0.30Å, and a maximum population size of 50 were established as docking boundaries. The internal electrostatic interaction (internal ES), sp2–sp2 rotations, and the internal H-bond interactions were recorded to assess the chemical affinity and connections of the mixes with the Bax, Bcl-2, Caspase-8, KDR1, and VEGF-A. Simplex development was established at maximum stages of 300 with a neighborhood distance feature of 1. Ten circles of docking were run, tested by post dock energy minimization applying the Nelder-Mead Simplex Minimization. The results were examined through Molegro Molecular Viewer and Discovery Studio, and the finest interrelating complex was designated from each database (25–27). **Figure 1B** shows the cavities of the targets (5W5X, 5JSN, 1I4E, 2QU5, and 5T89), which have the greatest potential for binding to ligands.

#### 2.1.3 ADME and toxicity forecasts

A successful medication candidate is defined by its good potential and, likewise, by sufficient ADME prediction. It is proposed that computational ADME utilization in a variety of *in vivo* and *in vitro* predictions leads to a decrease in the number of safety issues in the drug discovery procedure (28). In the medicine detection server (AdmetSAR), computational programs were utilized to evaluate the ADME and toxicity properties. AdmetSAR is a free and useful source in the ADMET prediction, and the properties of original chemical constituents are presented such as Absorption, Delivery, Digestion, and Elimination studies (http://lmmd.ecust.edu.cn/admetsar2/) (29–31).

### 2.2 *In vitro* examination

This study is an experimental study that has been performed in several stages on two human umbilical vein endothelial cell (HUVEC) cell lines as a control group and OVCAR-3 cells (ovarian cancer cells).

#### 2.2.1 Drug preparation

6-Gingerol (Catalog No. 23513-14-6) and cisplatin (CAS 15663-27) were purchased from Sigma-Aldrich Co. USA. Different concentrations of 6-gingerol as experimental groups and of cisplatin as positive control groups were prepared in sterile distilled water (25, 50, 100, and 200 µM).

#### 2.2.2 Cell culturing

HUVECs and OVCAR-3 cell lines were purchased from the Pasteur Institute of Iran (Tehran, Iran) and harvested in DMEM (Biosera, France) enriched with 10% fetal bovine serum (FBS) (Biosera, France) and 10,000 U/ml Pen/Strep (Thermo Fisher Scientific, USA) and incubated at 37°C in 5% of CO_2_.

#### 2.2.3 Cytotoxicity tests

HUVEC cell lines (5 × 10^4^) were counted and harvested in a 96-well plate and kept for 24 h. The plate's medium was replaced by fresh medium and 10 μl of different concentrations (25, 50, 100, and 200 μM) of 6-gingerol, cisplatin, and a mixture of them was added to each well. Treated cells were incubated at different time responses of 24, 48, and 72 h, and then 10 μl of MTT solution (Sigma-Aldrich, USA) and 5 mg/ml of MTT solution were added to each well and maintained for 3 h. This was followed by adding 100 μl of DMSO (Merk, Germany) to each well, which was kept in the dark for 1 h. The OD of absorbance was read at 490 nm by an ELISA reader (Bio Tek-ELX 800 Winooski, Vermont, USA). Fifty percent chemical concentrations (CC_50_) of the drug were considered using the probit test in the SPSS package.

#### 2.2.4 Selectivity index

The selectivity index (SI) as a measure of safety was calculated using the following equation: [SI = IC_50_ OVCAR-3/IC_50_ HUVECs] ≥10 to prove it is non-toxic. We also evaluated the combination index (CI) by the following formula: [CI = (D)/(D_x_)1 + (D)/(D_x_)2], where (D_x_)1 and (D_x_)2 are the concentration of the 6-gingerol and the cisplatin, respectively, used in the single treatment that was required to decrease the cell number by x% and (D) is the concentration of 6-gingerol in combination with the concentration of cisplatin that together decreased the cell number by x%. The CI value quantitatively defines synergism (CI < 1), additive effect (CI = 1), and antagonism (CI > 1). To determine the synergism activity of combination therapy, we determined the theoretic IC_50_ by the following method: [theoretic IC_50_ = (IC_50_ cisplatin/2) + (IC_50_ gingerol/2)].

#### 2.2.5 Cell cycle

The cells were counted, and 1×10^6^ cells were cultured in each well of a six-well plate. These steps were performed separately for both HUVECs and OVCAR-3 cell lines. After 72 h, viable cells were collected by trypsinization, and cell cycle analysis was performed after PI staining. Finally, the outcomes of cell nuclei stained with propidium iodide in its suspensions were analyzed by using a flow cytometer (Becton Dickinson, USA).

#### 2.2.6 Measurement of gene expression

The relative expression of apoptotic (Bax, Bcl-2, Caspase, and Apaf1) and angiogenetic (KDR, FLT1, and VEGF-A) mediator genes was determined by qPCR. Ovarian cancer cell lines were treated with 25, 50, and 100 μM of 6-gingerol, cisplatin, and a mixture of them and incubated for 48 h. Then, the cells’ total RNA was isolated with Trizol Reagenzien (Thermo Fisher Scientific, USA).

With the help of the High-Capacity cDNA Reverse Transcription Kit, corresponding DNA (cDNA) was created. The qPCR reaction was carried out using SYBR Green (Thermo Fisher Scientific, USA) and the Rotorgene Cycler system (Rotorgene 3000 cycler system). **Table 1A** demonstrates the template and control gene sequences. Gene expression was evaluated using the 2^−ΔΔCT^ method. The ΔCT was calculated by the following formulation: [ΔCT = CT (target) − CT].

**Table 1:** The specific primers and reference gene sequences for RT-qPCR in (A) *in vivo* and (B) *in vitro* examination.

	Gene	Forward sequence (5′–3′)	Reverse sequence (5′–3′)	Product size (bp)
	Bax	CCCGAGAGGTCTTTTTCCGAG	CCAGCCCATGATGGTTCTGAT	155
*A. In vitro*	Bcl-2	GGTGGGGTCATGTGTGTGG	CGGTTCAGGTACTCAGTCATCC	89
	Caspase-8	AGAGTCTGTGCCCAAATCAAC	GCTGCTTCTCTCTTTGCTGAA	78
	p53	CAGCACATGACGGAGGTTGT	TCATCCAAATACTCCACACGC	125
	FLT	CAGGCCCAGTTTCTGCCATT	TTCCAGCTCAGCGTGGTCGTA	82
	APAF1	AAG GTG GAG TAC CAC AGA GC	TCC ATG TAT GGT GAC CCA TCC	116
	KDR	CCA GCA AA CA GG GTCTGT	TGTCTGTGTCATCGGAGTGATATCC	87
	VEGF	CTACCTCCACCATGCCAAGT	GCA GTAGCTGCGCTGATAGA	109
	HPRT	CCTGGCGTCGTGATTAGTGAT	AGACGTTCAGTCCTGTCCATAA	131
	B2A	CATGTACGTTGCTATCCAGGC	CTCCTTAATGTCACGCACGAT	250
	GAPDH	ACAACTTTGGTATCGTGGAAGG	GCCATCACGCCACAGTTTC	101
*B. In vivo*	Bax	CCCGAGAGGTCTTTTTCCGAG	CCAGCCCATGATGGTTCTGAT	180
	Bcl-2	GGTGGGGTCATGTGTGTGG	CGGTTCAGGTACTCAGTCATCC	136
	APAF1	TTGCCAACCAGAGACATCAGAGG	TGCGGACGAACAACAACCAGACG	128
	TP53	ACCTGCACTTACTCCCCGGT	TCTTATAGACGGCCACGGCG	127
	KDR	GCCAACTCTATGGCAGAAGC	CTGAACACCATGCCACTGTC	86
	VEGF	CAATTGAGACCCTGGTGGAC	TCTCATCAGAGGCACACAGG	86
	B2M	GTGCTGGTGACCCTGGTG	CAGTTGAGGACGTTCTTGGTG	113
	HPRT	GATGAACAAGGTTACGACCTGGA	TATAGCCACCCTTGAGTACACAGAG	103
	GAPDH	CCTCTCTGGCAAAGTCCAAG	GGTCACGCTCCTGGAAGATA	176

### 2.3 *In vivo* examination

#### 2.3.1 YSM assay

Ross 308 fertile eggs with a weight of 55 ± 0.5 g were purchased from Simorgh Co. (Kerman, Iran) and kept under standard conditions (37°C ± 1°C in 75% humidity). To evaluate the anti-angiogenic activity of 6-gingerol and cisplatin, the yolk sac membrane (YSM) assay was conducted using a mixture of them in a chick embryo model. At first, fertile eggshells were cleaned and a little spot was created on the shell; then, 50 µl of 6-gingerol, cisplatin, and their combination (as the experimental group) and PBS (as control) were injected into the embryo. In the next 24 and 48 h, the drugs were re-injected into the eggs repeatedly. After each injection, the eggshells were cleaned and closed with molten paraffin and incubated under the same standard conditions. On day 4 (22–24 stages of the Hamburger–Hamilton growth stage), the eggshell membranes broke and were studied under a stereomicroscope (Luxeo 4D Stereozoom Microscope, Labomed, CA, USA), and high-quality images (4,000 × 3,000 pixels) were taken for YSM analysis by using ImageJ^®^ 1.48 (National Institutes of Health, Bethesda, Maryland, USA) and MATLAB^®^ (Mathworks Matlab R2015a) software. Vascular density was computed with these data.

#### 2.3.2 Molecular assay

Relative angiogenetic (KDR and VEGF) and apoptotic (Bax, Bcl-2, TP53, and Apaf1) mediator gene expression changes in chick embryos that were treated with 6-gingerol, cisplatin, and their mixture were evaluated by real-time qPCR. The entire RNA isolated from the extraembryonic membrane was extracted with Trizol Reagenzien (Thermo Fisher Scientific, USA) and the concentration of RNA was read by a Nanodrop device (Thermo Scientific, Wilmington, DE). After the synthesis of cDNA by using the High-Capacity cDNA Reverse Transcription Kit (Thermo Fisher Scientific, USA), the qPCR assay was carried out using a SYBR Green assay (SYBR Premix Ex Taq TM II, Takara Bio, Inc., Shiga, Japan).

**Table 1B** lists the specific primers and common gene combinations. Forty cycles of magnification were carried out after the initial step of 95°C for 1 min. Each cycle lasted for 10 s at 9°C for DNA denaturation, 30 s at 60°C for annealing, and 30 s at 72°C as an extension. The expression profile was examined using the predefined standard genes.

#### 2.3.3 Histopathological assessment

The chicken embryo specimens were fixed in a 10% formalin solution first. The formalin-fixed paraffin-embedded samples were processed using the microtome (Slee-Germany) in 4-µm sections and thereby stained with routine hematoxylin and eosin (H&E) for assessment of histopathological changes. After that, selected samples were stained by immunohistochemical (IHC) apoptosis and angiogenesis markers including Bax (Zytomed Germany, code number: 502-17990), Bcl-2 (mouse monoclonal antibody; code number: PDMO16- lot No. H147 from the US), and CD34. Positively stained cells were counted in 10 fields, and their means show the Bcl-2, Bax, and CD34 expression levels.

### 2.4 Statistical analysis

Statistical analyses were performed using IBM^®^ SPSS^®^ (V.20) and GraphPad Prism (V.8.0) software. All data were analyzed by one-way ANOVA and paired ***t***-test analysis. Statistical significance was set at *p* < 0.05. All experiments were replicated at least three times.

## 3 Results

### 3.1 *In silico* modeling

#### 3.1.1 MVD molecular docking

**Figure 2:**
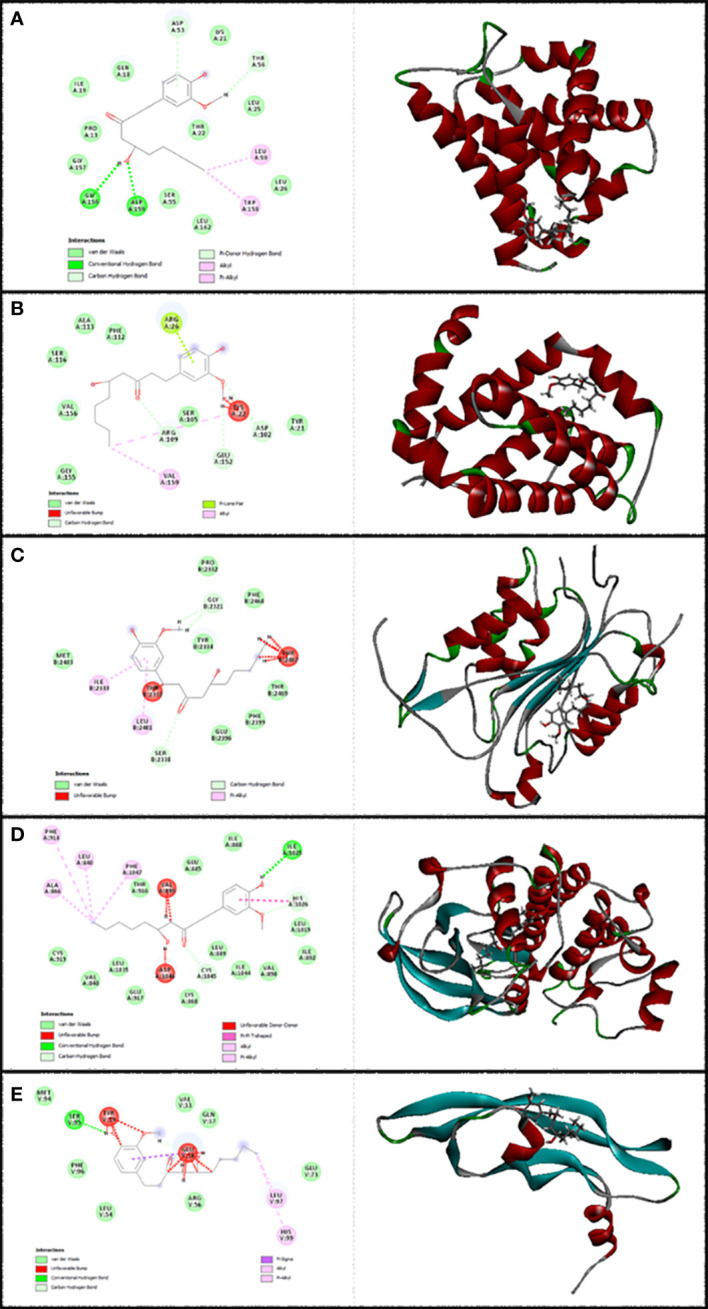
Illustration of the finest score docking solution of the 6-gingerol ligands and **(A)** Bax, **(B)** Bcl-2, **(C)** Caspase-8, **(D)** KDR1, and **(E)** VEGF-A receptor with the designated crystal construction of 5W5X, 5JSN, 1I4E, 2QU5, and 5T89, respectively, and a ligand map with various chemical bonds courtesy of Discovery Studio.

**Figure 3:**
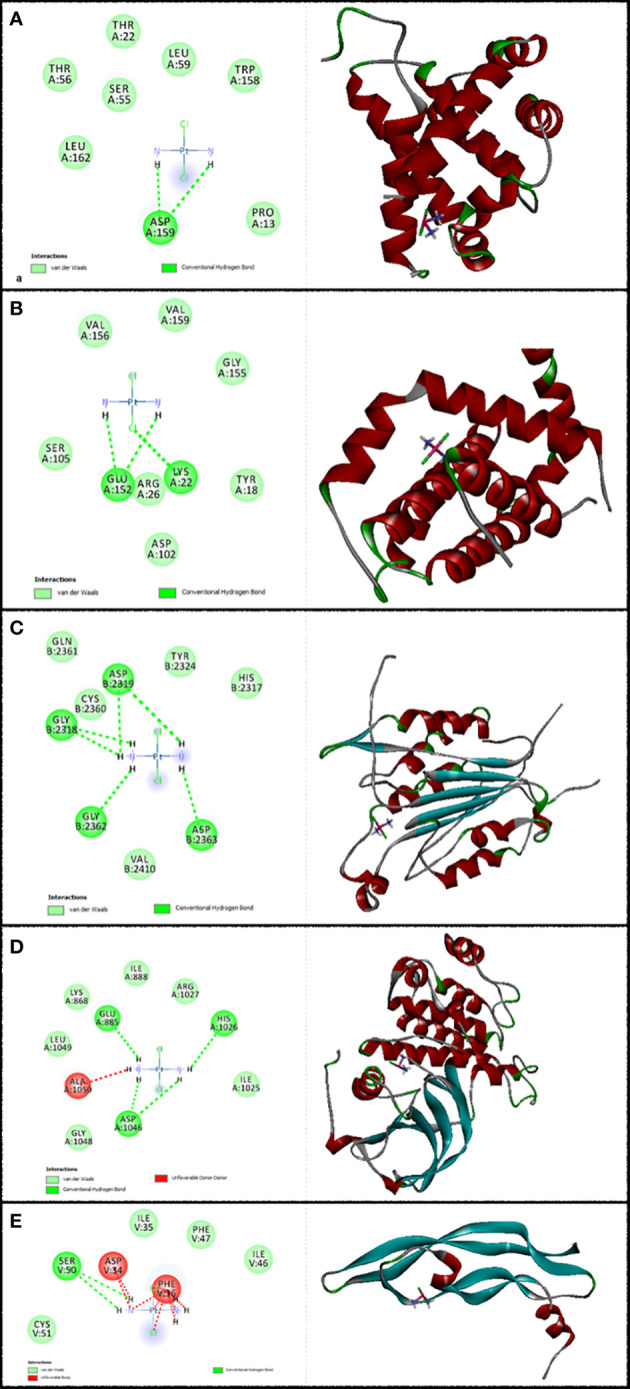
Illustration of the finest score docking solution of the cisplatin ligands and **(A)** Bax, **(B)** Bcl-2, **(C)** Caspase-8, **(D)** KDR1, and **(E)** VEGF-A receptor with the designated crystal construction of 5W5X, 5JSN, 1I4E, 2QU5, and 5T89, respectively, and a ligand map with various chemical bonds courtesy of Discovery Studio.

**Figure 4:**
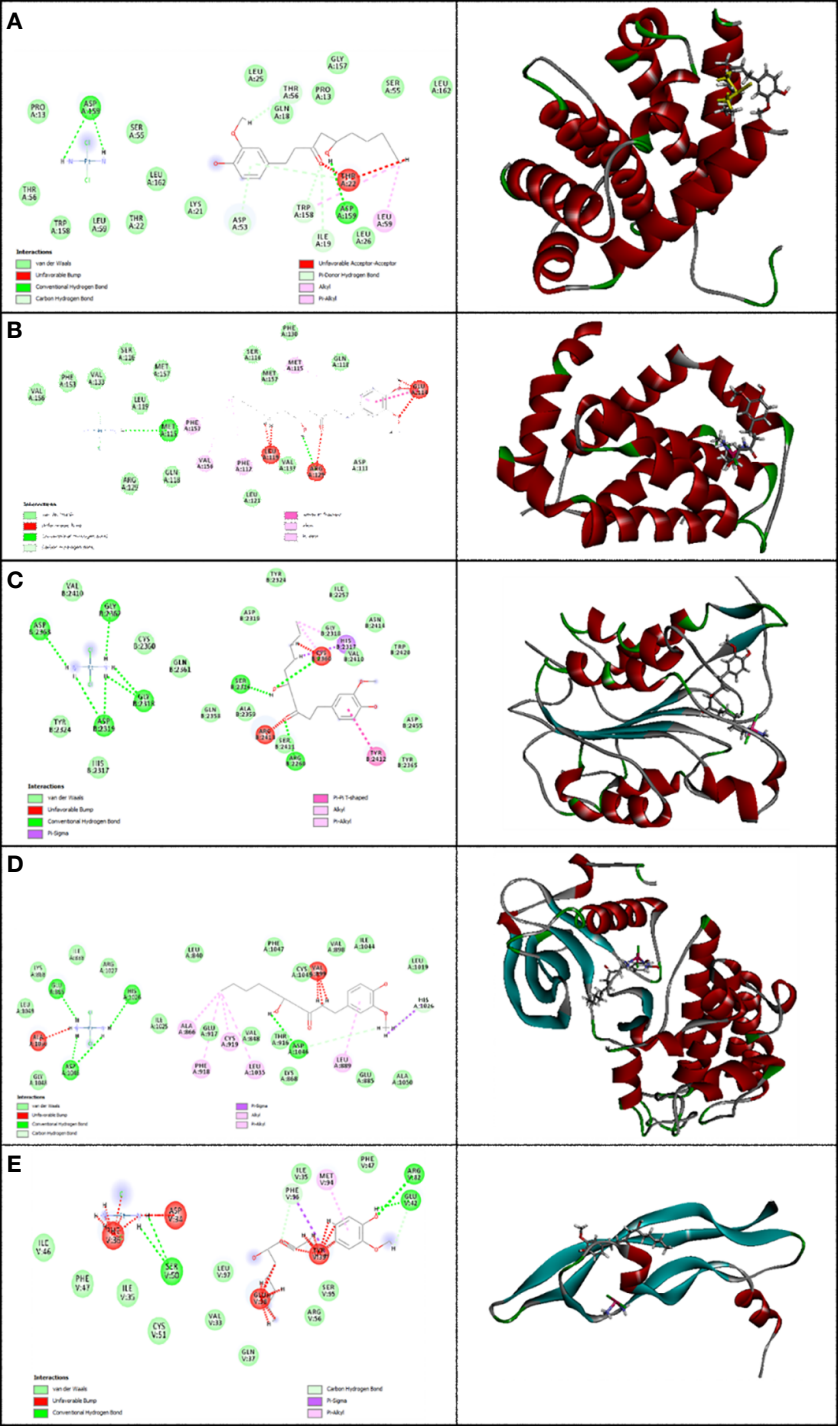
Representation of the finest score docking solution of the mixture of the two drugs and best ligands and **(A)** Bax, **(B)** Bcl-2, **(C)** Caspase-8, **(D)** KDR1, and **(E)** VEGF-A receptor with the designated crystal structure of 5W5X, 5JSN, 1I4E, 2QU5, and 5T89, respectively, and a ligand map with various chemical bonds courtesy of Discovery Studio.

In this research work, the focus is on the interactions of 6-gingerol, cisplatin, and the combination of these drugs. MVD molecular docking conformation and analysis showed the 6-gingerol, cisplatin, and combination forms of the three drug reagents that interacted with Bax, Bcl-2, Caspase-8, VEGF-A, and KDR1. Free total energy or MolDock Score values were subject to negative energy values, indicating that the binding events of the complexes were spontaneous. **Table 2** displays the docked configuration of the complexes with the related parameters. Schematic molecular docking results and ligand maps of structures and the 5W5X, 5JSN, 1I4E, 2QU5, and 5T89 targets are shown in **Figures 2**–**4**.

**Table 2:** Parameters from the interaction between 6-gingerol, cisplatin, and their mixture with angiogenetic and apoptotic mediators.

Compound	Docking score for 6-gingerol	Docking score for cisplatin	Docking Score for the mixture of the drugs
Bax	−103.917	−44.78	−146.78
Bcl-2	−103.36	−41.01	−119.152
Caspase-8	−106.16	−48.31	−156.54
KDR1	−121.37	−39.86	−153.52
VEGF-A	−83.42	−41.36	−142.16

The MolDock Score values for 6-gingerol were −103.917, −103.36, −106.16, −121.37, and −83.42 where they were docked to Bax, Bcl-2, Caspase-8, KDR1, and VEGF-A receptors, respectively.

The resulting data from molecular docking is presented in **Figure 2A** where 6-gingerol forms van der Waals, conventional hydrogen bonds, carbon hydrogen bonds, pi-donor hydrogen bonds, alkyl, and pi-alkyl with amino acids of the Bax using Asp 53, Thr 56, Gly 156, Asp 159, Leu 59, and Trp 158. Furthermore, 6-gingerol forms van der Waals forces, carbon hydrogen bonds, pi-lone pair, alkyl, and unfavorable bumps with amino acid residues (Arg 109, Asp 102, Glu 152, Arg 26, Val 159, and Lys 22) of the Bax (**Figure 2B**). In **Figure 2C**, the 6-gingerol molecule forms van der Waals, carbon hydrogen bonds, pi-alkyl, and unfavorable bumps with amino acid residues of Caspase-8 receptor including Gly 2331, Ser 2338, Ile 2333, Leu 2401, Thr 2337, and Thr 2467. Moreover, 6-gingerol was stabilized by KDR1 using van der Waals, conventional hydrogen bond, carbon hydrogen bonds, alkyl, pi-alkyl, pi–pi T-shaped, unfavorable donor–donor, and unfavorable bumps with the following amino acid residues: Ile 2025, His 1026, Cys 1045, Leu 840, Ala 866, Phe 918, Phe 1047, Val 899, and Asp 1046 (**Figure 2D**). Subsequently, the residues Glu 38, Ser 95, Leu 97, His 99, and Tyr 39 of VEGF-A interact with 6-gingerol as seen in **Figure 2E** by van der Waals, conventional hydrogen bond, carbon hydrogen bonds, alkyl, pi-alkyl, pi-sigma, and unfavorable bumps.

Values of the MolDock Score for cisplatin were −44.78, −41.01, −48.31, −39.86, and −41.36, which were docked to Bax, Bcl-2, Caspase-8, KDR1, and VEGF-A receptors, respectively. Also, cisplatin forms van der Waals and conventional hydrogen bonds with the amino acid residue (Asp 159) of the Bax (**Figure 3A**). **Figure 3B** shows that cisplatin forms van der Waals and conventional hydrogen bonds with amino acids of the Bcl-2 using Lys 22, Arg 26, and Glu 152. Additionally, cisplatin binds to the Caspase-8 receptor with a binding site consisting of amino acid residues Gly 2318, Asp 2319, Gly 2362, and Asp 2363 with van der Waals and conventional hydrogen bond (**Figure 3C**). Cisplatin forms van der Waals, conventional hydrogen bond, and unfavorable bumps with amino acids of the KDR1 using Glu 885, His 1026, Asp 1046, and Ala 1050 (**Figure 3D**). Cisplatin binds into the dynamic spot of VEGF-A with a binding site consisting of amino acid residues like Ser 50, Asp 34, and Phe 36 with van der Waals, conventional hydrogen bond, and unfavorable bumps (**Figure 3E**).

Furthermore, the MolDock Score values of Bax Bcl-2, Caspase-8, KDR1 and VEGF-A for the mixture of these two compounds were −146.78, −119.152, −156.54, −153.52, and −142.16, respectively.

Subsequently, **Figure 4A** shows that the mixture of these two compounds forms van der Waals, conventional hydrogen bond, carbon hydrogen bonds, alkyl, pi-alkyl, pi-donor hydrogen bonds, unfavorable acceptor–acceptor, and unfavorable bumps with amino acids of the Bax when targeting Thr 56, Asp 53, Asp 159, Ile 19, Trp 158, Leu 59, and Thr 22. From the docking analysis, its outcome data expressed clearly in **Figure 4B** that the mixture of these two compounds formed van der Waals, conventional hydrogen bonds, carbon hydrogen bonds, amide-pi stacked hydrogen bonds, alkyl, pi-alkyl, and unfavorable bumps with amino acids of the Bcl-2 against Met 115, Arg129, Asp 111, Phe 112, Phe 153, Val 159, Glu 114, and Leu 119. Also, residues Ser 2316, Gly 2318, Asp 2319, Gly 2362, Asp 2363, Cys 2360, Ser 2411, Tyr 2412, and Arg 2413 of Caspase-8 interacted with the two compounds’ mixture as displayed in **Figure 4C** by van der Waals, conventional hydrogen bond, pi-sigma, alkyl, pi-alkyl, pi–pi T-shaped, and unfavorable bumps. At the same time, we need to point out that cisplatin formed van der Waals, conventional hydrogen bonds, carbon hydrogen bonds, pi-sigma, alkyl, pi-alkyl, and unfavorable bumps of the KDR1 against Glu 885, His 1026, Asp 1046, Ala 866, Phe 819, Cys 919, Leu 1035, and Ala 1050 (**Figure 4D**). In addition, the mixture was stabilized by VEGF-A using van der Waals, conventional hydrogen bond, carbon hydrogen bonds, pi-sigma, pi-alkyl, and unfavorable bumps with amino acid residues Phe 96, Ser 50, Glu 42, Arg 82, Met 94, Asp 34, Phe 36, Glu 38, and Tyr 39 (shown in **Figure 4E**).

This study indicates that 6-gingerol and cisplatin interacted with apoptotic and antiapoptotic proteins of Bax, Bcl-2, Caspase-8, KDR1, and VEGF-A. Consequently, 6-gingerol was more effective than cisplatin. Subsequently, there is the ultimate confirmation that the binding affinity of 6-gingerol is better than that of cisplatin, while that of the mixture of the two drugs is the best with Bax, Bcl-2, Caspase-8, KDR1, and VEGF-A.

#### 3.1.2 ADMET prediction

Before experimental approaches, ADMET prediction (Chemical Absorption, Distribution, Metabolism, Excretion, and Toxicity analysis) is employed to indicate the pharmacokinetics of molecules (31).

ADMET properties showed that cisplatin had a less human intestinal absorption (HIA) score than 6-gingerol, which implies that the compound could have less intestinal absorption against oral administration. The greatest penetration within the blood–brain barrier (BBB) is seen for cisplatin. While it appears to indicate the efflux by P-glycoprotein (P-GP), both compounds’ measurement results show them as a substrate and inhibitor of P-GP. Likewise, in terms of metabolism, 6-gingerol and cisplatin were substrates (but non-inhibitors) of the CYP450 microsomal enzyme. A non-inhibitor of CYP450 demonstrates that the compounds will not prohibit the biotransformation of the drug metabolized by the CYP450 enzyme. The test of AMES toxicity is used to determine the mutagenic molecule. 6-Gingerol and cisplatin indicated a negative AMES toxicity test, which implies that these are not mutagenic. Furthermore, the carcinogenic terms showed that the molecules were not carcinogenic. Subsequently, 6-gingerol included lower oral toxicity than cisplatin. Likewise, considerable data were estimated by ADMET prediction, such as the LD_50_ dose in a rat model. In comparison, a compound with a greater LD_50_ dose is less deadly than that having a lower LD_50_ dose. It has been defined from ADMET results that 6-gingerol had less LD_50_ and was more toxic compared to cisplatin (2.4106 versus 2.7419, respectively). Likewise, the greater value of the log S, the lower the solubility, which would reduce the absorption (32). Consequently, cisplatin with a lower log S has better absorption than 6-gingerol, indicating its low bioavailability, which makes it more resistant to oxidation and hydrolysis, and thus, with improved stability, improved protection toward degradation of the cisplatin molecules, and increased bioavailability compared to 6-gingerol (33). **Supplementary 1** represents the different ADMET parameters gained from the admetSAR tool.

### 3.2 In vitro

#### 3.2.1 Cytotoxicity

**Figure 5:**
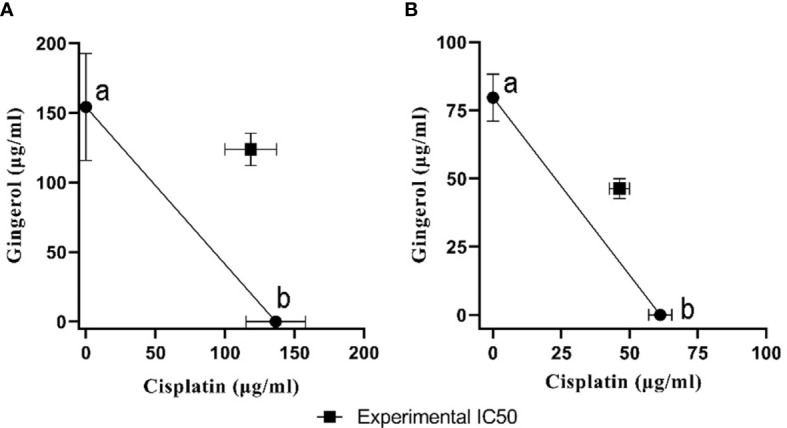
The isobologram analysis of the effects of the drug combination of cisplatin and 6-gingerol. **(A)** In HUVECs, foci a and b displayed the IC_50_ value of cisplatin (136.52 ± 21.36 µM) and 6-gingerol (154.2 ± 38.43 µM), respectively. Theoretical IC_50_ was 145.36 µM and our experimental IC_50_ was 118.6 ± 18.52 µM. **(B)** In OVCAR-3 cells, foci a and b displayed the IC_50_ value of cisplatin (61.23 ± 4.22 µM) and 6-gingerol (79.66 ± 8.63 µM), respectively. Theoretical IC_50_ was 70.46 µM and our experimental IC_50_ was 46.33 ± 3.68 µM. Statistical analysis revealed that there was a significant difference between experimental IC_50_ and theoretical IC_50_ (*p* < 0.001).

To evaluate the cytotoxicity and effect of 6-gingerol alone, cisplatin alone, and their combination on HUVECs and OVCAR-3, the colorimetric method was used. The results showed that combining these two has a far more significant effect than each drug alone: in HUVECs, 118.6 ± 18.52 in mixed compared to 136.52 ± 21.36 and 154.2 ± 38.43 for cisplatin and 6-gingerol, respectively; in OVCAR-3, 46.33 ± 3.68 in mixed compared to 61.23 ± 4.22 and 154.2 ± 38.43 for cisplatin and 6-gingerol, respectively (**Table 3**). The isobologram analysis results are demonstrated in **Figure 5**.

**Table 3:** Evaluating the IC_50_ values and selective index (SI) of cisplatin, gingerol, and combination therapy.

Drugs	IC_50_ HUVECs	IC_50_ OVCAR-3	SI (OVCAR-3/HUVECs)
Cisplatin	136.52 ± 21.36	61.23 ± 4.22	2.22
6-gingerol	154.2 ± 38.43	79.66 ± 8.63	1.93
Mix	118.6 ± 18.52	46.33 ± 3.68	2.55

#### 3.2.2 6-Gingerol, cisplatin, and their combination-induced apoptosis

**Figure 6:**
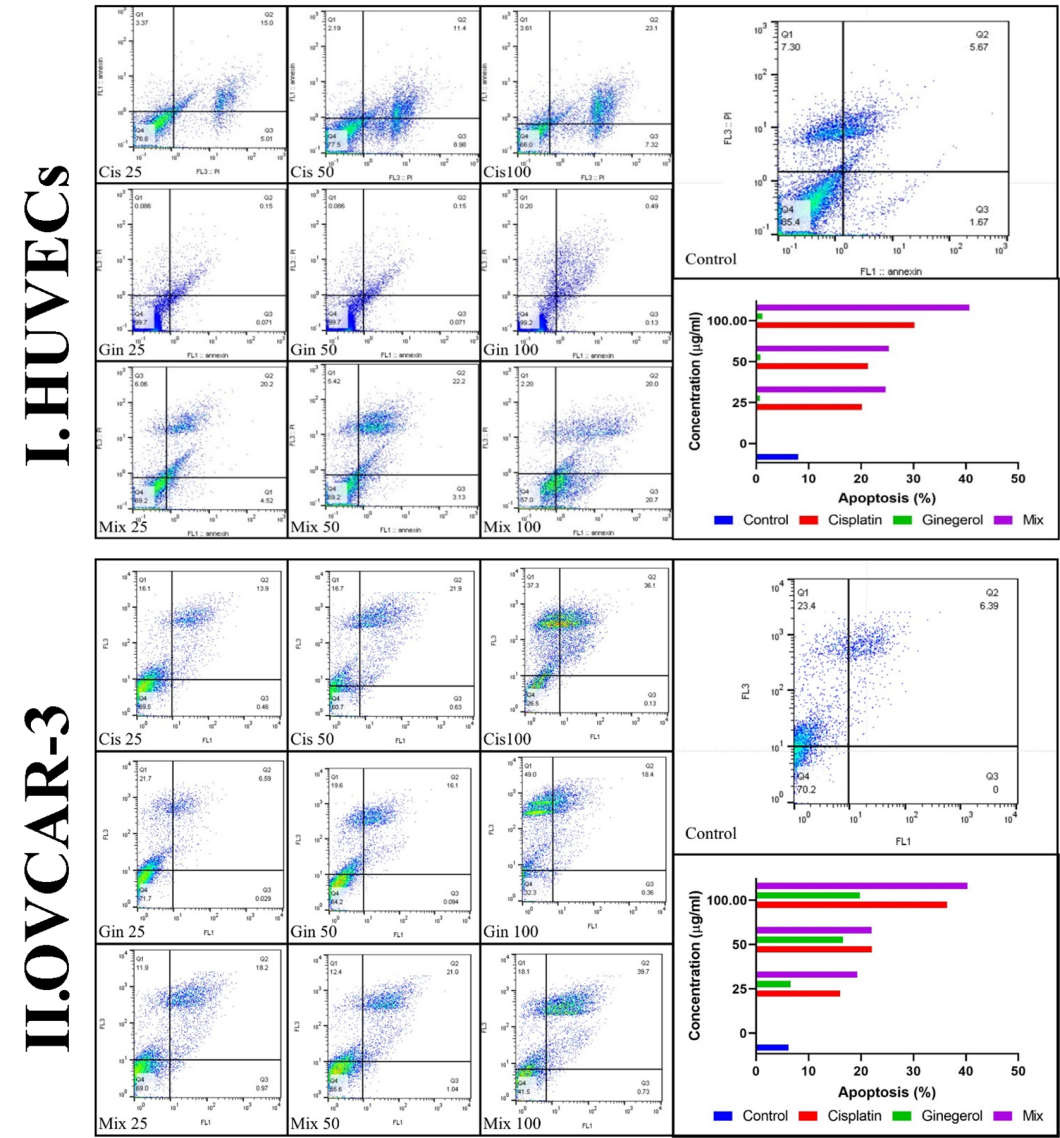
Characteristics of apoptosis and necrosis of **(I)** HUVECs and **(II)** the OVCAR-3 cell line treated with different concentrations of cisplatin, 6-gingerol, and the combination therapy.

Treatment of HUVECs and OVCAR-3 with 6-gingerol alone, cisplatin alone, and their combination led to apoptosis. All concentrations of the three treated sets presented significant differences relative to the negative control group (*p* < 0.001). Cisplatin significantly increased the apoptotic level compared to 6-gingerol. Also, all 6-gingerol plus cisplatin combined concentrations showed significantly higher apoptosis and decreased necrosis (**Figure 6**).

#### 3.2.3 Cell cycle

**Figure 7:**
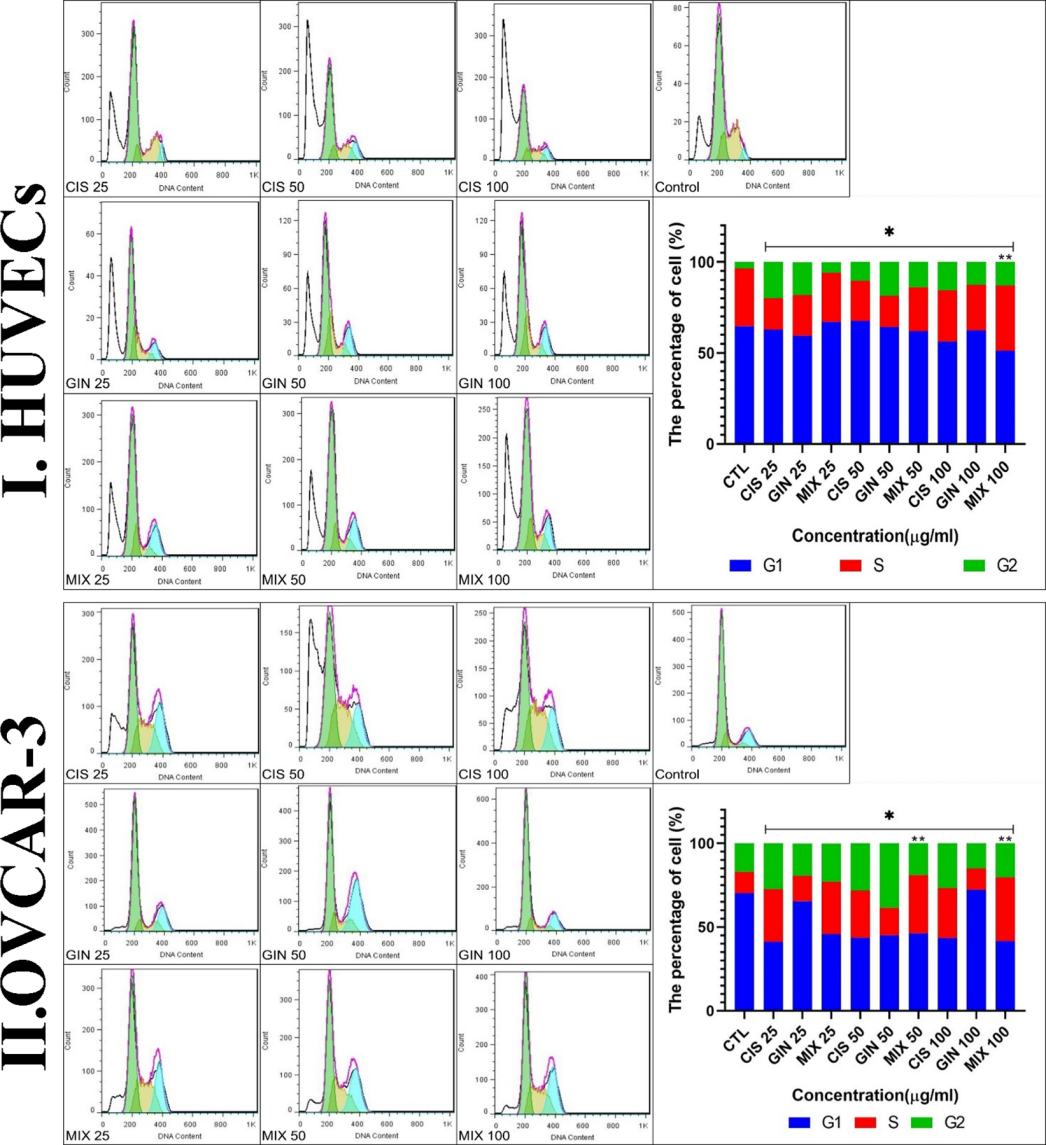
Cell cycle characteristics of **(I)** HUVECs and **(II)** the cell line treated with different concentrations of cisplatin, 6-gingerol, and the combination therapy [*significant difference with the control group (*p* < 0.001), **significant difference between drug combination therapy and cisplatin (*p* < 0.001)].

Cell cycle analysis showed that in the treatment of HUVECs and OVCAR-3 cell lines by different concentrations of cisplatin, 6-gingerol, and the combination therapy, the duration of the S cycle increases with increasing concentrations of drugs, indicating a prolongation of cell division time, which, in turn, slows down cell division. The results showed that this rate was significantly higher at a dose of 100 μg/ml combination therapy than in the cisplatin treatment group alone (**Figure 7**).

#### 3.2.4 Gene expression

**Figure 8:**
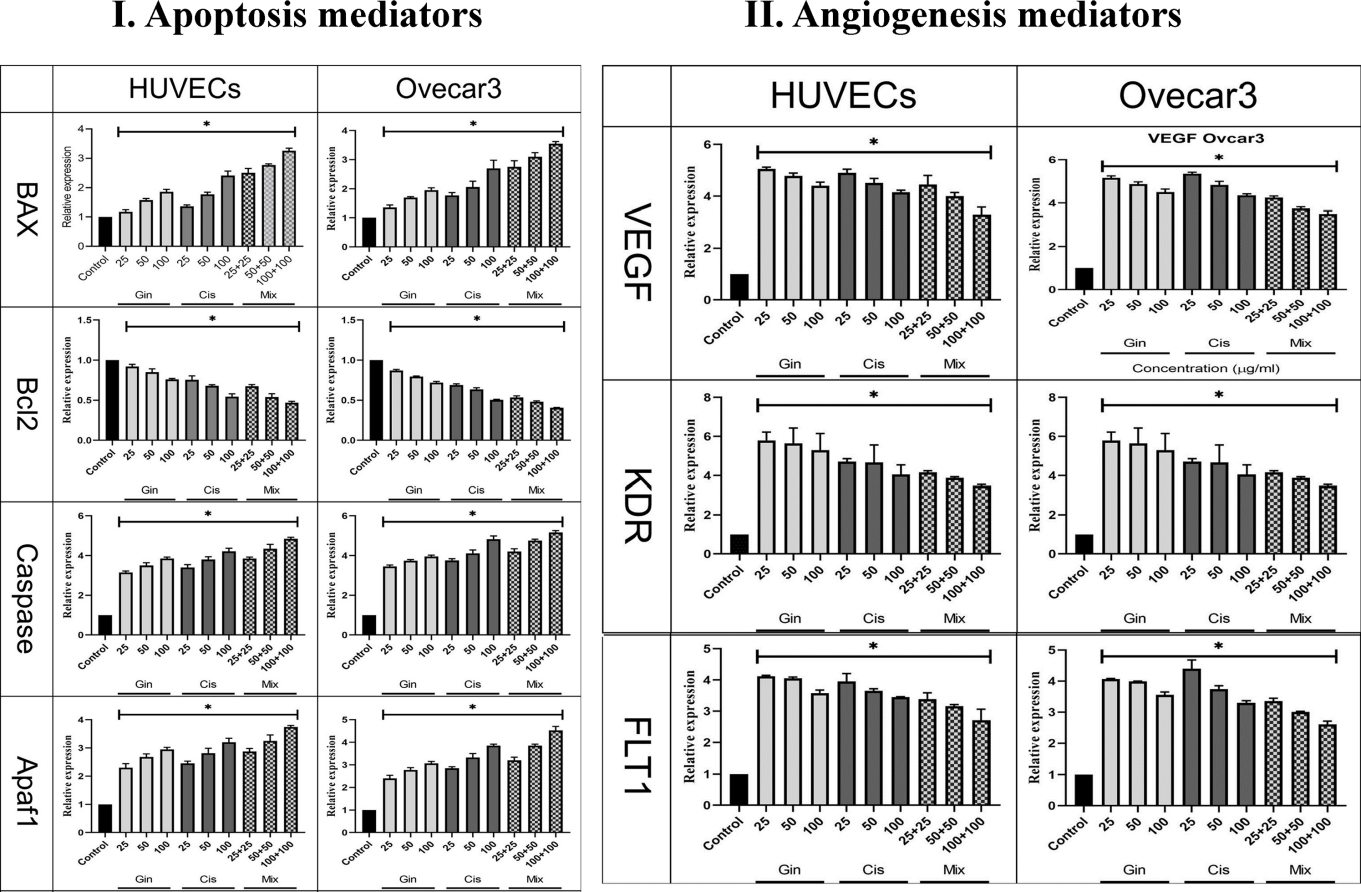
Evaluation of **(I)** apoptotic (Bax, Bcl-2, Caspase, p53, and Apaf1) and **(II)** angiogenetic (KDR, FLT1, and VEGF) gene expression in HUVECs and OVCAR-3 cell lines treated with different concentrations of cisplatin, 6-gingerol, and the combination therapy [*significant difference with the control group (*p* < 0.001)].

The study of apoptotic (Bax, Bcl-2, Caspase-8, p53, and Apaf1) and angiogenetic (KDR, FLT1, and VEGF-A) gene expression showed that in terms of the cumulative concentrations of drugs in cell lines, the expression levels were significantly elevated compared to the control group (*p* < 0.001). This significant increase in combination treatment was also detected when compared to cisplatin therapy (*p* < 0.05) (**Figure 8**).

### 3.3 In vivo

#### 3.3.1 Vascular density

**Figure 9:**
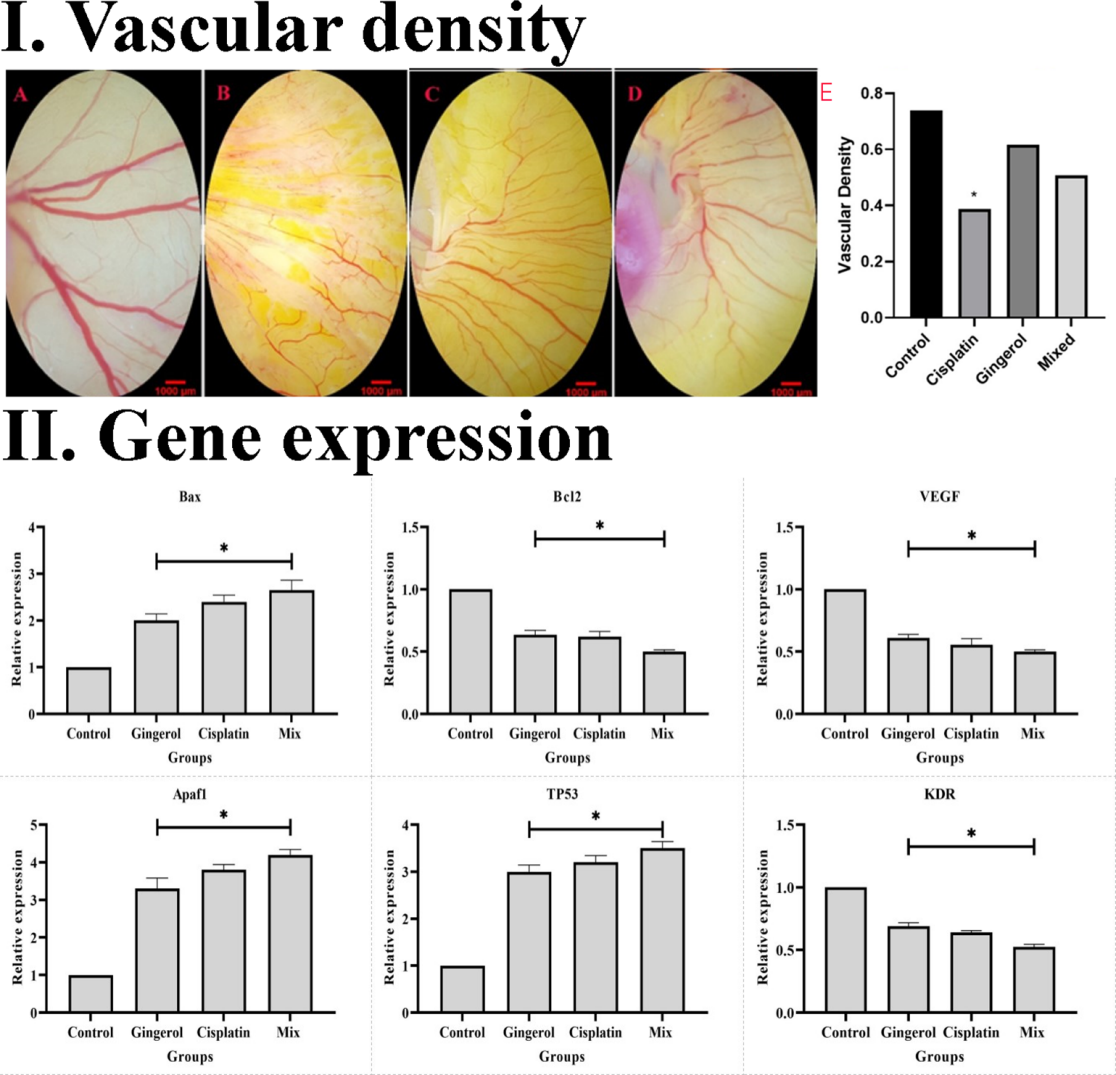
**(I)** Effect of cisplatin, 6-gingerol, and the combination therapy on the chick embryo’s blood vessels. **(A)** Control group, **(B)** cisplatin, **(C)** 6-gingerol, **(D)** mixture, and **(E)** vascular density (error bars show mean ± standard error; *p < 0.05 compared to the control group). **(II)** Effect of cisplatin, 6-gingerol, and the combination therapy on the apoptotic (Bax, Bcl-2, Apaf1, and TP53) and angiogenetic (VEGF and KDR) mediator gene expression changes treated embryos compared to controls. The expression levels were normalized to GAPDH and HPRT and calibrated to controls (error bars show mean ± standard error; *p < 0.05).

The effect of the 6-gingerol, cisplatin, and the combination therapy on the chick’s YSM at 24, 48, and 72 h of primary growth is given. The vascular density of the treated embryos’ vasculature significantly decreased in both 6-gingerol- and cisplatin-treated groups. According to statistical analysis, 6-gingerol-treated embryos had less vascular density than cisplatin embryos (**Figures 9 IA–E**).

#### 3.3.2 Gene expression

The results revealed that the expression profile of apoptotic gene markers (Bax, Apaf1, and TP53) was significantly increased in cisplatin, 6-gingerol, and combination therapy compared to the untreated control group (*p* < 0.05). The Bcl-2 gene expression as an apoptotic mediator in all treated groups decreased compared to the untreated control groups (*p* < 0.05). Angiogenesis mediator genes including VEGF and KDR in all treated groups significantly decreased in comparison to the untreated control group (*p* < 0.05) (**Figure 9 II**).

#### 3.3.3 Immunohistochemistry assay

**Figure 10:**
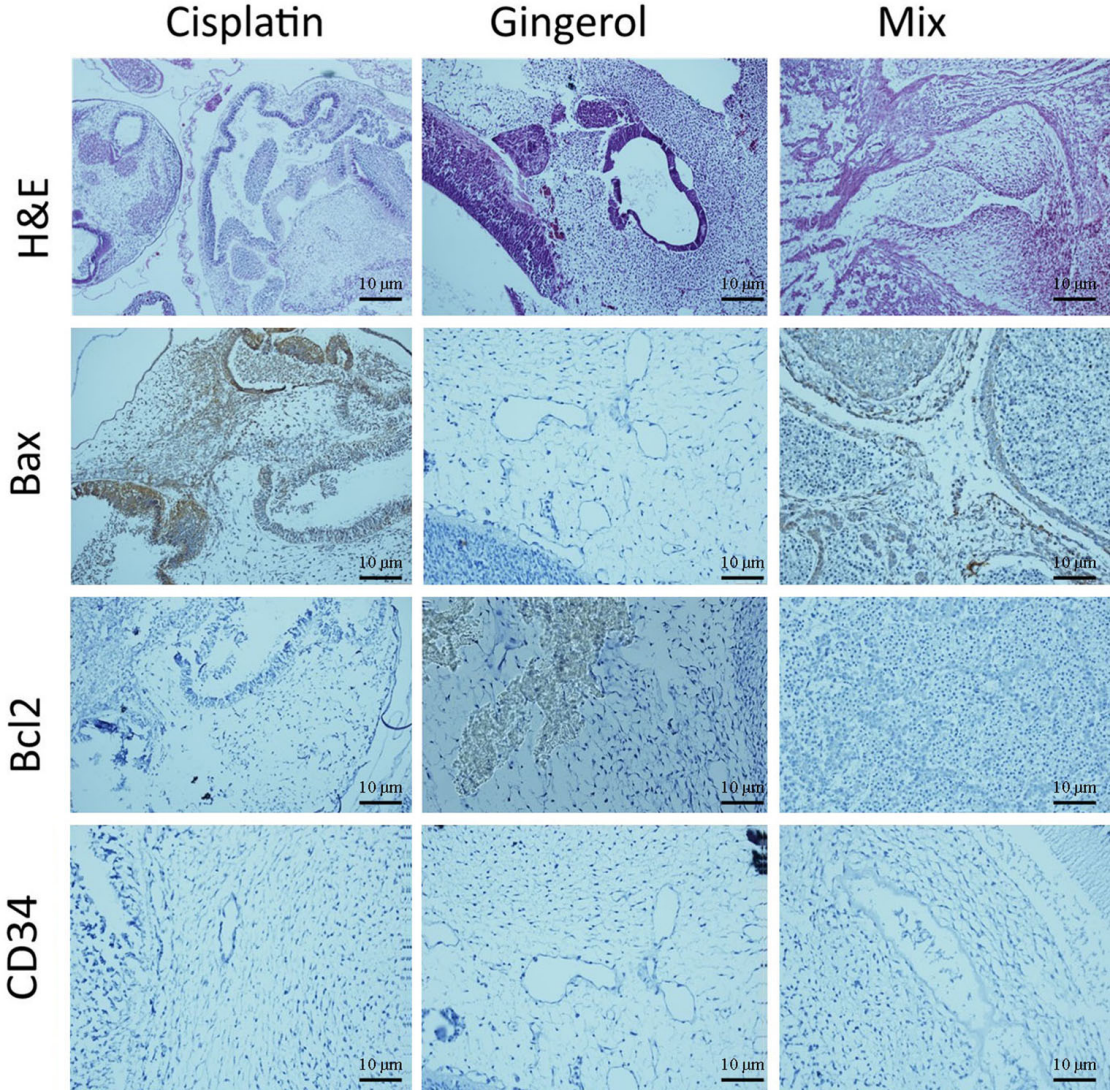
Histopathological changes in H&E and IHC study [apoptotic (Bax and Bcl-2) and angiogenetic (CD34) markers] of the chick embryo treated with cisplatin, 6-gingerol, and the combination therapy.

From a histopathological point of view and by comparing different patterns with each other and the control group, we concluded that cisplatin had teratogenic effects by decreasing the growth and development of most of the three germ layer cells and induced tissue atrophy in their absence. 6-Gingerol had no major effects on embryogenesis, which was mostly similar to normal control. However, in the combination of 6-gingerol and cisplatin, it seemed that the teratogenic effects of cisplatin decreased markedly, but still observed a dispersed disruption of the embryogenesis. These changes were evaluated by H&E staining and also immunohistochemical staining for Bax, which were more prominent in cisplatin. Bcl-2 and CD34 had fewer changes in 6-gingerol and combination therapy, in order of frequency (**Figure 10**).

In the H&E assay, results show that cisplatin has degenerative to necrotic changes in embryonic tissue as a result of disruption of the integrity of structural cells. For 6-gingerol, both mesenchymal and epithelial cells and even neural tube components seemed to react normally with non-damaged embryonic growth and development, and the heart and its chambers that were subjected to mixed therapy seem to have a well-preserved architecture with less likely degenerative changes compared with the cisplatin group.

The Bax marker, an apoptotic mediator, showed that cisplatin induced multifocal strong positive staining of both embryonic mesenchymal and epithelial cells. 6-Gingerol showed weak positive staining in embryonic tissues, and the combination therapy showed moderate positive cytoplasmic staining in different embryonic tissues. The Bcl-2 marker, another apoptotic mediator, showed that cisplatin influences damaged embryonic tissues with negative cytoplasmic staining in embryonic mesenchymal and epithelial tissues. In 6-gingerol, focally positive staining of mesenchymal cells was noted. In the combination therapy, dispersed cytoplasmic staining of embryonic tissue was noted. The CD34 marker, an angiogenetic mediator, showed that cisplatin and vascular channels seemed to have opened, but atrophic lumen and sloughing of endothelial cells were noted. In 6-gingerol, vessels seemed to open without damage to the components of the vessel wall. In combination therapy, the lumen of the vessel was opened but focal sloughing of endothelial cells and degeneration of the vessel wall components were noted.

## 4 Discussion

Herbal products are traditionally used to treat disease, and in current clinical trials, more than 50% of medicines are from natural resources (34). 6-Gingerol is the most active metabolite derived from ginger, which is an anti-angiogenic and apoptotic drug (35). Due to the anticancer properties of 6-gingerol, and the risks of cisplatin, in this study, we compared the outcome of 6-gingerol, cisplatin, and their combination therapy on the process of angiogenesis and apoptosis of ovarian cancer cells and HUVEC.

Cytotoxicity analysis of this study shows that combination therapy was increased compared to cisplatin or 6-gingerol in both cell lines. CI and isobologram analysis show the combination therapy in both cell lines had an antagonistic activity. Nevertheless, various combinations of treatment besides cisplatin displayed degrees of antagonism based on the CI values or isobologram analysis (36). Although the combination treatment of 6-gingerol and cisplatin has shown the antagonistic effects of the drugs, other methods used in this study confirm the reduction of the adverse effects of the combined treatment compared to the treatment with cisplatin.

A similar study by Kapoor et al. (19) in 2016 evaluated the antitumor activity of 6-gingerol on human oral cell lines (SCC4 and KB) and cervical malignancy (HeLa) with or without cortmanine, rapamycin, and cisplatin. Moreover, in the MTT assay evaluation, the percentage of viable cells in the mixture of 6-gingerol with cortmanine and cisplatin was lower than in the treatment with 6-gingerol or cisplatin and cortmanine alone. Although the toxicity of 6-gingerol and cisplatin has not been studied in any other study, the results of similar studies (33, 37) in this field show that the cytotoxicity of 6-gingerol increases with increasing dose. The study by Kim et al. (38) had no toxicity of 6-gingerol at doses below 20 μg. As shown in the results of the colorimetric analysis of our study, cytotoxicity at doses below 25 μg was lower in 6-gingerol than in the mixed group, and cisplatin was more toxic. According to these results, it can be said that, in addition to enhancing the performance of cisplatin, combined treatment also reduces its side effects to a great extent.

Flow cytometry findings demonstrated that the apoptotic value of the two combined drugs was higher while the necrosis was lower than cisplatin in both OVCAR-3 cells and HUVECs alone, and increased with enhanced concentrations. In the case of 6-gingerol in HUVECs, the apoptosis rate was low due to the antioxidant nature of the drug and the resistance of these cells. The apoptotic effect of 6-gingerol was more significant in OVCAR-3 cells than in HUVECs. In support of the above results, we can point out several similar studies are in line with the present study results. For example, the results of the study from Kapoor et al. (19) showed that the combination therapy of 6-gingerol with cortmanine, rapamycin, and cisplatin significantly increased the rate of apoptosis in human oral cancer cells (SCC4 and KB) and cervical cancer (HeLa). Numerous studies (39–41) have shown that combination therapy with expected agents sensitizes HeLa cells to lower concentrations of cisplatin. Rastogi et al. (18) conducted a study to evaluate this effect. Their study showed that a concentration of 50 μM 6-gingerol sensitizes cervical malignant cells to 2.5 μM cisplatin. This combination promoted the apoptotic cells following 24 h of treatment. Similarly, in a study by Nipin et al., 6-gingerol increased early apoptosis (42).

The activity of the 6-gingerol and cisplatin combination on the cell cycle showed that the S phase at a concentration of 100 µg/L in both OVCAR-3 cells and HUVECs had the shortest time. There are several studies on the apoptosis of various cancer cells in the vicinity of 6-gingerol, although a small number, such as the present study, have examined the effect of 6-gingerol and cisplatin alone or their combination therapy. In 2021, Nipin et al. (42) examined the action of 6-gingerol on the apoptosis of breast cancer cells. According to their study, 6-gingerol stopped the cell cycle in the G0/G1 phase. This study showed that 6-gingerol could induce early and late apoptosis by failing to induce DNA repair and long-term cessation of the cell cycle.

Another study (33) showed that treatment with 6-gingerol inhibited HPV-positive cervical cancer cell proliferation by reactivating p53, increasing oxidative stress, and inducing DNA impairment related to G2/M cell cycle arrest and apoptosis.

The findings showed a concentration of 50 μM 6-gingerol sensitized cervical cancer cells to 2.5 μM cisplatin. This mixture augmented the apoptotic level in cells treated for 24 h. The cell cycle analysis showed that when the combination of 6-gingerol and cisplatin was used, a significant accumulation of cells in the G2/M cell cycle phase occurred. In addition, it exhibited an increase in apoptotic cells treated with 6-gingerol and cisplatin compared to each drug treatment alone. Overall, these results confirmed that 6-gingerol enhances the antiproliferative effects of cisplatin by inducing DNA damage due to oxidative stress and cell death in cervical cancer cells and a potent stimulus for p53 reactivation in cervical HPV cancer cells. It is positive and the results can be used as a chemical sensitizer for conventional chemotherapy drugs such as cisplatin.

The study carried out by Kapoor et al. (19) examined the apoptotic and anticancer properties of 6-gingerol with and without cisplatin in the treatment of oral cancer cell lines (Scc4 and KB) and a cervical cancer cell line (HeLa). This study showed that when 6-gingerol and cisplatin were coupled, their effect was significantly increased by 50% against the apoptosis of the above cancer cells, compared to individual drugs alone. Furthermore, treatment with 6-gingerol or with cisplatin alone had better therapeutic results in all three cancer cell lines.

The possibility of 6-gingerol combined with cisplatin as a novel treatment for gastric cancer was examined by Luo et al. (43). The mixture of 6-gingerol and cisplatin repressed cell viability and enhanced cell cycle arrest in the G1 phase compared with cisplatin alone. Combination treatment lowered cyclin D1, cyclin A2, matrix metalloproteinase-9, p-PI3K, AKT, and p-AKT protein expression; raised P21 P27 mRNA levels; and hindered the capacity of cells to relocate and migrate. This study demonstrated that 6-gingerol improves gastric cancer cells’ susceptibility to the chemotherapy drug cisplatin, and the processes involved in migratory inhibition, suppression of invasion, and G1 phase arrest *via* the PI3K/AKT signaling pathway.

Another investigation by Park et al. (44) found that 6-gingerol inhibited cell growth by stopping the cell cycle in the G1 phase in each cell line of pancreatic cancer and by preventing cells from entering phase S. This stops the growth and proliferation of cancer cells.

Regarding the effect of 6-gingerol, cisplatin, and their combination on the expression of oncogenes and genes for the induction of apoptosis and cellular angiogenesis, the findings of our study revealed that the combination of these two drugs, as against each alone, in both OVCAR-3 and HUVEC cell lines increased the expression of cells that induce cell apoptosis such as Apaf1, Bax, Caspase-8, and p53 and decreased the expression of Bcl-2 (as oncogenes) and VEGF, KDR, and FLT1 (as cells that induce angiogenesis). The analysis of gene expression results in our study is consistent with several similar studies in this field, each of which is discussed separately.

Protease 1-activating apoptosis factor (Apaf1) is a gene that encodes a cytoplasmic protein that is one of the major gateways to the cell death regulatory network. In the present study, the expression of the Apaf1 gene showed that by promoting the concentration of drugs in both cell lines, the expression of the Apaf1 gene also increased, which showed a significant difference from the control group. This significant increase was also observed in combination therapy compared to cisplatin therapy. The above results are consistent with the results of several similar studies, which are given below.

In the study of Nigam et al. (17), 6-gingerol was evaluated for its anti-apoptotic potential in human epidermoid carcinoma (A431). Treatment with 6-gingerol showed significant cytotoxicity, as it inhibited the proliferation of A431 cells. Mediated production of ROS was identified. Increased ROS decreased mitochondrial membrane potential (MMP) and triggered subsequent apoptosis. Treatment with 6-gingerol also resulted in high regulation of cytochrome c and Apaf1, followed by caspase cascade and apoptosis.

The tumor suppressor gene p53 enhances Bax gene expression, and this protein plays an essential role in p53-dependent apoptosis. Bcl-2 is an oncogene that inhibits apoptosis and cancer progression. A drug that can increase Bax expression and decrease Bcl-2 expression can prevent cancer growth. Examination of Bax gene expression in our study showed that with rising drug concentrations in both cell lines, the expression of the Bax gene also rose, demonstrating a considerable departure from the control group. This difference between combination therapy and cisplatin treatment was also detected. Regarding the expression of the Bcl-2 gene, the results of our study showed that, in both cell lines, by increasing the concentration of drugs, the expression of the Bcl-2 gene decreased, which shows a significant difference from the control group; this significant decrease in combination therapy was also observed in comparison to cisplatin treatment alone.

The results of similar studies in this field support the above finding. In the study of Nipin et al. (42), 6-gingerol increased Bax expression and decreased Bcl-2 expression, followed by loss of membrane potential and subsequent formation of pores in the mitochondrial membrane of breast cancer cells, which indicates that a positive effect of 6-gingerol is involved in inducing apoptosis in cancer cells. Luo et al. (43) investigated the anti-apoptotic effects of 6-gingerol on gastric adenocarcinoma (AGS) cells. The results showed that abnormalities in MMP were associated with the deregulation of the Bax/Bcl-2 ratio at the protein level, which resulted in positive regulation of cytochrome-c, resulting in a caspase cascade and subsequent induction of apoptosis. Chakraborty et al. (45) looked at how 6-gingerol affected the apoptosis of HeLa cells. The results showed that 6-gingerol therapy decreased the overexpression of NFk, AKT, and Bcl-2 genes in cancer cells. On the other hand, 6-gingerol-treated cells showed an increase in the expression of TNF, Bax, and cytochrome c. They concluded that 6-gingerol might bind to DNA and cause cell death *via* apoptosis and autophagy mediated by caspase.

The p53 gene is the most well-known tumor-blocking gene, mutating in more than 50% of human cancers. This vital role in genomic stability and tumor suppression is mainly involved in inducing cell cycle arrest, aging, programmed cell death, and inhibition of angiogenesis. The study of p53 gene expression showed that, in both cell lines, by elevating the concentration of drugs, the expression of the p53 gene was also augmented, which shows a significant difference from the control group. This significant increase in combination therapy compared to treatment with cisplatin was also detected. A review of similar studies showed that 6-gingerol induced apoptosis in various cancers by increasing p53 expression. In the study by Liu et al. (46), 6-gingerol increased p53 levels and decreased the Bcl-2/Bax ratio, and, of course, endometrial cancer cell death and mitochondrial membrane potential were significantly increased in endometrial cancer cell lines after exposure. Exposure was reduced and induced. Also, in a study by Park et al. (44), 6-gingerol increased p53 expression and induced apoptosis of pancreatic cancer cells. In a study by Rastogi et al. (18), it was found that 6-gingerol inhibited proteasome and oxidative stress by increasing p53, which stopped apoptosis and cell division in breast cancer cell lines. A tumor suppressor gene induces cell apoptosis by increasing its expression and preventing cancer cell proliferation. With rising medication concentrations in both cell lines in the current study, the expression of the Caspase-8 gene also rose, demonstrating a substantial difference from the control group. This large increase in combination treatment is also related to the factor to be seen after receiving cisplatin therapy.

In the present investigation, the apoptotic effect of 6-gingerol, cisplatin, and their combination demonstrated in embryonic vessels was assessed through *in silico* and *in vivo* studies. In this regard, we discuss various highlights of the findings regarding vascular changes and the interactions of 6-gingerol, cisplatin, and their combination with proteins, which are associated with apoptosis.

In the current paper, we applied a docking assay to clarify some details about the apoptotic effect of 6-gingerol, cisplatin, and their combination demonstrated in vessels. Currently, docking is considered a useful technique to study the interactions between receptors and ligands; thus, it is applied in various molecular investigations (47, 48). It is well known that the Bcl-2 family members are important targets for apoptotic and anti-apoptotic factors (49, 50).

Combination treatment demonstrated promising *in silico* results, which are revealed by their substantial scoring roles and increased protein–ligand interface binding energy. The *in silico* ADMET results indicated that combination treatment is promising for the improvement of a particular, safe, and efficient anticancer process. The conclusion of the analysis provided a significant development for combination therapy as a great anticancer agent in general.

To confirm our prediction; the toxicity of 6-gingerol, cisplatin, and the combination treatment was also accessed *via in vivo* (YSM) assay. Due to the considerable decreases in vessel area and diameter seen in the YSM vessels, it can be concluded that 6-gingerol and cisplatin demonstrated a negative effect on the embryonic vasculature. Based on these results, we suggest the use of the combination treatment (compared to cisplatin alone) on the fetus.

To the best of our knowledge, this is the first study that targets the different aspects of 6-gingerol, cisplatin, and combination treatment toxicity with a chick embryo model.

In our study, vascular analysis of YSM shows that 6-gingerol, cisplatin, and the combination treatment damaged the embryonic vessels. The method used to assess the apoptotic effect of 6-gingerol, cisplatin, and the combination treatment was to calculate the MCA in the obtained images. Until now, this technique has been used in various research (51, 52). Another highlight to be explained is the significant change in the expression of Bax and Bcl-2 proteins following 6-gingerol, cisplatin, and the combination treatment. These altered expressions in apoptotic–regulator components can make a link between 6-gingerol, cisplatin, and the combination treatment and vascular defect that was seen in the current study. The pathways or mechanisms by which 6-gingerol, cisplatin, and the combination treatment cause toxic effects on blood vessels are not fully understood, but according to our results, it can be suggested that the vascular toxicity of 6-gingerol, cisplatin, and the combination treatment is associated with the induction of apoptotic-signaling pathways. The IHC results also confirmed the changes in the expressions of Bax and Bcl-2 in the 6-gingerol-, cisplatin-, and the combination treatment-exposed embryos.

## 5 Conclusion

The results of this study showed that cisplatin as the first line of ovarian cancer treatment can prevent the progression and proliferation of cancer cells, but it also causes some complications for the cells. 6-gingerol can reduce the side effects of this drug and increases its effectiveness when combined with cisplatin. On the other hand, because of the anti-nausea properties of ginger, it is possible to use this herbal substance widely and in combination with cisplatin drugs by presenting specific drug protocols.

Finally, other studies in this field could be performed *in vivo* and in later stages of human trials to provide the basis for progression in the administration of this drug combination to improve the quality of life for patients with ovarian cancer.

## Data availability statement

The original contributions presented in the study are included in the article/**Supplementary Material**. Further inquiries can be directed to the corresponding authors.

## Ethics statement

The animal study was reviewed and approved by the present study and was performed based on the suggested European Ethical Guidelines by the care of animals in experimental investigations. It was approved by ethical committee of Kerman University of Medical Sciences (Project No. 97000719) and ethic code IR.KMU.REC.1397.154.

## Author contributions

ZS: original draft preparation, data validation, review, and editing. AK: original draft preparation and supervision. EP: original draft preparation and supervision. EM: software and molecular docking analysis. ES, ARK, HT, and SS: methodology. SD: visitation. GR: review and editing. All authors contributed to the article and approved the submitted version.
