# Supplementary material for: The inhibitory effect of 6-gingerol and cisplatin on ovarian cancer and antitumor activity: In silico, in vitro, and in vivo
Source: Front Oncol. 2023 Mar 3;13:1098429. doi: 10.3389/fonc.2023.1098429 (PMC10020515; doi:10.3389/fonc.2023.1098429)
Supplement: Supplementary file 1 [file Table_1.docx]

**Supplementary 1** - ADMET properties of the compounds

| Model | Compound | | |  |
| --- | --- | --- | --- | --- |
| Absorption | **6-gingerol** | | **Cisplatin** | |
| Blood-Brain Barrier | BBB- | 0.6072 | BBB- | 0.9554 |
| Human Intestinal Absorption | HIA- | 0.9805 | HIA- | 0.9571 |
| Caco-2 Permeability | Caco2- | 0.6843 | Caco2- | 0.5594 |
| P-glycoprotein Substrate | Substrate | 0.7319 | Substrate | 0.8682 |
| P-glycoprotein Inhibitor | Inhibitor | 0.8440 | Inhibitor | 0.9658 |
|  | Non-inhibitor | 0.5172 | Non-inhibitor | 0.9743 |
| Renal Organic Cation Transporter | Non-inhibitor | 0.8858 | Non-inhibitor | 0.9168 |
| Distribution | | | | |
| Subcellular localization | Mitochondria | 0.9052 | Mitochondria | 0.7330 |
| Metabolism | | | | |
| CYP450 2C9 Substrate | Non-substrate | 0.8432 | Non-substrate | 0.7550 |
| CYP450 2D6 Substrate | Non-substrate | 0.8002 | Non-substrate | 0.7760 |
| CYP450 3A4 Substrate | Substrate | 0.5724 | Substrate | 0.7295 |
| CYP450 1A2 Inhibitor | Non-inhibitor | 0.6632 | Non-inhibitor | 0.7573 |
| CYP450 2C9 Inhibitor | Non-inhibitor | 0.8278 | Non-inhibitor | 0.7708 |
| CYP450 2D6 Inhibitor | Non-inhibitor | 0.7926 | Non-inhibitor | 0.8899 |
| CYP450 2C19 Inhibitor | Non-inhibitor | 0.6350 | Non-inhibitor | 0.7636 |
| CYP450 3A4 Inhibitor | Non-inhibitor | 0.5902 | Non-inhibitor | 0.8736 |
| CYP Inhibitory Promiscuity | Low CYP Inhibitory Promiscuity | 0.8581 | Low CYP Inhibitory Promiscuity | 0.8683 |
| Toxicity | | | | |
| AMES Toxicity | Non-AMES toxic | 0.9403 | Non-AMES toxic | 0.9611 |
| Carcinogens | Non-carcinogens | 0.5451 | Non-carcinogens | 0.9337 |
| Fish Toxicity | High FHMT | 0.7697 | High FHMT | 0.6079 |
| Tetrahymena Pyriformis Toxicity | High TPT | 0.9121 | High TPT | 0.5183 |
| Honey Bee Toxicity | High HBT | 0.9832 | High HBT | 0.8902 |
| Biodegradation | Not ready biodegradable | 0.9997 | Not ready biodegradable | 0.9123 |
| Acute Oral Toxicity | II | 0.6474 | III | 0.5276 |
| ADMET Predicted Profile --- Regression | | | | |
| Aqueous solubility | LogS | [3.2344](http://lmmd.ecust.edu.cn/admetsar1/predict/?smiles=CCCCCC(CC(=O)CCC1=CC(=C(C=C1)O)OC)O&action=A) | LogS | 2.0431 |
| Caco-2 Permeability | LogPapp, cm/s | [1.0418](http://lmmd.ecust.edu.cn/admetsar1/predict/?smiles=CCCCCC(CC(=O)CCC1=CC(=C(C=C1)O)OC)O&action=A) | LogPapp, cm/s | 0.6792 |
| Rat Acute Toxicity | LD50, mol/kg | [2.4106](http://lmmd.ecust.edu.cn/admetsar1/predict/?smiles=CCCCCC(CC(=O)CCC1=CC(=C(C=C1)O)OC)O&action=A) | LD50, mol/kg | 2.7419 |
